# The distribution and chemosensory responses of pharyngeal taste buds in the sea lamprey *Petromyzon marinus*

**DOI:** 10.1007/s00359-024-01708-3

**Published:** 2024-07-30

**Authors:** Hasan Polat, Gianfranco Grande, Zeenat Aurangzeb, Huiming Zhang, Gheylen Daghfous, Réjean Dubuc, Barbara Zielinski

**Affiliations:** 1 Department of Integrative Biology, University of Windsor, Windsor, ON Canada; 2 Department of Biomedical Sciences, University of Windsor, Windsor, ON Canada; 3 Groupe de Recherche en Activité Physique Adaptée, Département des Sciences de l’activité physique, Université du Québec à Montréal, Montréal, QC Canada; 4 Department of Neurosciences, Université de Montréal, Montréal, QC Canada

**Keywords:** Lampreys, Pharynx, Gustation, Electrophysiology, Chemoreception, Taste buds

## Abstract

Little is known about the chemosensory system of gustation in sea lampreys, basal jawless vertebrates that feed voraciously on live prey. The objective of this study was to investigate taste bud distribution and chemosensory responses along the length of the pharynx in the sea lamprey. Scanning electron microscopy and immunocytochemistry revealed taste buds and associated axons at all six lateral pharyngeal locations between the seven pairs of internal gill pores. The most rostral pharyngeal region contained more and larger taste buds than the most caudal region. Taste receptor cell responses were recorded to sweet, bitter, amino acids and the bile acid taurocholic acid, as well as to adenosine triphosphate. Similar chemosensory responses were observed at all six pharyngeal locations with taste buds. Overall, this study shows prominent taste buds and taste receptor cell activity in the seven pharyngeal regions of the sea lamprey.

## Introduction

**Fig. 1:**
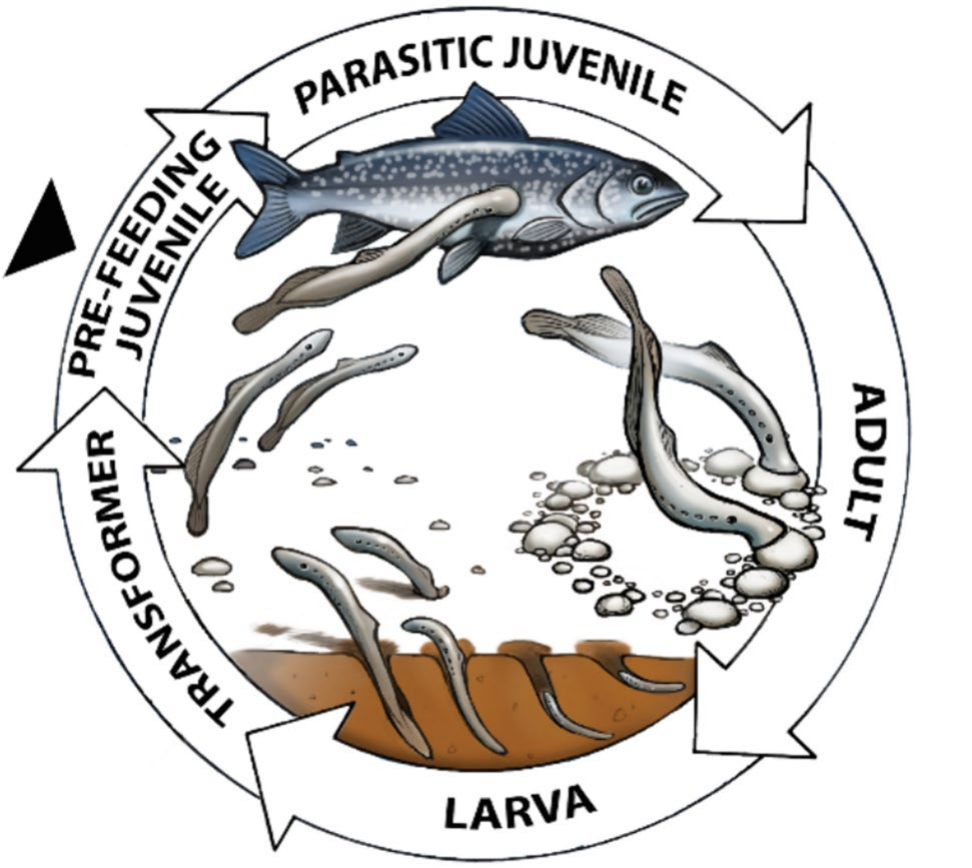
Life cycle of the sea lamprey (*P. marinus*). The life stages are larva, transformer, pre-feeding juvenile, parasitic juvenile and adult (Clemens 2019; Evans et al. 2021). The drawing was adapted from the Great Lakes Fishery Commission website. The large black arrowhead points to the pre-feeding juvenile stage that was investigated in this study. The larval stage filter feeds and burrows in detritus and sandy substrate. During metamorphosis, the transformers stop feeding as the body undergoes metamorphic changes. The pre-feeding juvenile stage has completed metamorphosis and moves from silty habitat to open waters where the parasitic juvenile stage attaches onto prey and feeds on the blood and flesh of the prey, eventually killing the host fish (Farmer 1980). The pre-feeding juveniles are approximately 13 cm long (Evans et al. 2021) and the size range for the parasitic juvenile stage is 78 to 120 cm in length (Potter et al. 2015). During the adult stage the sea lampreys do not consume food, but migrate to spawning grounds, construct nests and then spawn

In many animal species, the chemosensory modality of gustation (taste) enables the perception of sweet, sour, bitter, salty and savory (amino acids) during feeding (Finger 1997). The chemosensory neuroepithelial cells associated with taste are aggregated into specialized structures called taste buds and are located within mound-like epithelial papillae (Finger 1997; Michel 2006; Uchida 1980). In mammals, these neuroepithelial taste cells generate action potentials in response to taste stimuli (Avenet and Lindemann 1991; Cummings et al. 1993; Gilbertson et al. 1992). It is generally accepted that lampreys are the oldest extant vertebrates that possess true taste buds (Finger 1997; Northcutt 2004). In these basal vertebrates with evolutionary lineage that diverged from the main vertebrate line approximately 500 million years ago (Hedges 2001), taste buds are located on the lateral surface of the pharynx, between adjacent gill pores in larval and adult stages (Baatrup 1983a, b; Barreiro-Iglesias et al. 2008; 2010 Finger 1997; Mallat 1979; Northcutt 2004). Neural activity recordings from pharyngeal regions containing taste buds in larval stage European brook lampreys (*Lampetra planeri*) have demonstrated chemosensory responses to canonical taste stimuli, including bitter, sweet, sour, amino acid and salty tastants (Baatrup 1985). While the larvae of all lampreys are burrowing, microphagous and filter feeding, some lamprey species, including the European brook lamprey, do not feed following metamorphosis. However other species, including the sea lamprey (*Petromyzon marinus)* feed by attaching onto prey and consuming flesh and bodily fluid during the post-metamorphic (juvenile) stage (Evans et al. 2021; Farmer 1980; Kleerekoper and Mogensen 1963; Hardisty 1979; Gill et al. 2003) (Fig. 1). The body length increases substantially from approximately 13 cm in the pre-feeding juvenile stage (Evans et al. 2021) to the 78–120 cm range for parasitic juvenile sea lampreys (Potter et al. 2015). This feeding has impacted at the ecosystem level, as fish populations declined substantially due to predation by sea lampreys in the Great Lakes of North America during the twentieth century, when invasive sea lampreys populated this region (Gaden et al. 2021). There have been no studies of gustation in lampreys that feed on host fish. The goal of the present study is to investigate the anatomical distribution and chemoreception of taste buds in post-metamorphic sea lampreys. Upon the completion of metamorphosis, this prefeeding juvenile stage swims to open water where feeding commences (Evans et al. 2021). Field observations of this stage have documented aggressive feeding (Bird et al. 1994). The current study investigates chemosensory taste responses to prey cues such as the bile acid taurodeoxycholic acid (TDCA), since bile acids are taste stimulants for teleost fish (Giaquinto and Hara 2008; Rolen and Caprio 2008). The nucleotide adenosine triphosphate (ATP) was tested, as ATP gustation activates feeding in blood-sucking mosquitoes (Galun et al. 1963; Jové et al. 2020). Sweet and amino acid gustatory stimuli were also tested.

## Materials and methods

### Experimental animals

All experiments were performed in accordance with the guidelines of the University of Windsor Animal Care Committee and the Canadian Council on Animal Care. Pre-feeding juvenile sea lampreys (Evans et al. 2021) were obtained from the Hammond Bay Biological Station (United States Geological Survey Hammond Bay, Michigan, USA), transported to the Animal Care Facility at the University of Windsor and held in dechlorinated water under static renewal conditions at 6 °C from November to July. While the pre-feeding juvenile stage does not require feeding, these lampreys did feed on live fish in our lab. For this study, 60 pre-feeding juveniles were investigated (110–178 mm long; 1.6–3.6 g). These were collected in the autumn, as the pre-feeding juveniles were moving from streams into open water. Seven parasitic juvenile lampreys were examined for pharyngeal taste bud responses in this study as well. These ranged from 150–490 mm in length and from 16.3–401 g in weight.

### Scanning electron microscopy

Pre-feeding juvenile sea lampreys (n = 2; 129, 133 mm, 1.66, 1.73 g) were anesthetized using dechlorinated water containing 0.5 g/L of tricaine methane sulphonate (MS-222). To isolate the pharynx, the lampreys were cut transversely slightly caudal to the seventh external gill pore and rostral to the first external gill pore. Dissected samples were immediately placed into 5% glutaraldehyde fixative in 0.1 M sodium cacodylate buffer, then further dissected with a cut through the ventral midline to expose the pharyngeal cavity. The specimens were then placed in separate vials of 2% osmium tetroxide solution in 0.1 M sodium cacodylate buffer for 30 min then carefully removed and dehydrated through an ethanol series of 30 min for, 50% ethanol, 70% ethanol, 85% ethanol, 95% ethanol, followed by three changes of absolute (100%) alcohol washes. The samples were stored in absolute ethanol until critical point drying. Samples were critical point dried using the (Denton DCP-1) and gold sputter coated (Denton TSC Desk V) at the University of Guelph (Advanced Analysis center), then placed in a desiccation chamber for storage. The samples were examined under an FEI Quanta 200 Environmental Scanning Electron Microscope with an EDAX Octane Plus SDD detector at the University of Windsor Great Lakes Institute of Environmental Research (GLIER).

### Immunohistochemistry and data analysis

Acetylated tubulin (AT) immunocytochemistry was used to locate the exact position, distribution, and size of taste buds in different pharyngeal regions. Barreiro-Iglesias et al. (2008) used this cellular labelling technique to investigate the structure and composition of taste buds in sea lamprey. While the taste cells within vertebrate taste buds are microvillous (Boudroit and Reutter 2001; Tichy 1991; Uchida 1980), lamprey taste cells are ciliated (Baatrup 1983a, b; Barreiro-Iglesias et al. 2008). Pre-feeding juvenile (n = 7, 140–145 mm, 2.4–3.6 g) lampreys were anesthetized using dechlorinated water solutions of 0.5 g/L of tricaine methanesulphonate (MS-222). The length and weights were 110 mm (not weighed), 138 mm (2.4 g), 140 mm (2.5 g), 140 mm (2.6 g), 140 mm (not weighed), 14.8 cm (3.8 g). After dissection, the pharynx was harvested from the rest of the lamprey body and put into 4% paraformaldehyde in 0.1 M phosphate buffer, pH 7.4 overnight. After fixation, the tissue was cryoprotected in 20% sucrose and 30% sucrose in phosphate buffered saline and cryosectioned on a Leica CM3050S cryostat at a thickness of 20 μm. Sectioned tissue were immersed in 100% acetone for 10 min at-20 °C, followed by a wash for 10 min with 0.1 M phosphate buffered saline. Cryosections were incubated in primary antibody 1:2500 anti-acetylated tubulin raised in mouse (1:1000, Sigma-Aldrich Cat# T7451 RRID: AB_609894) plus the blocking buffer with 5% normal goat serum for three days at 4 °C temperature. The acetylated tubulin antibody was used to immunolabel taste cells in the pharyngeal cavity (Barreiro-Iglesias et al. 2008). Slides were then washed with 0.1 M PBS every 3 min for three cycles then incubated in secondary antibody (1:250 goat anti-mouse 488 IgG Sigma Aldrich Oakville ON, A11004 lot# 1,698,376) for two days at 4 °C. Slides were washed in 0.1 M phosphate buffed saline every 30 min for four cycles and then counterstained with DAPI and cover slipped with Vectashield mounting medium (H-1200, Vector Laboratories, Burlingame, CA). We applied criteria used by Barreiro-Iglesias et al. (2008) for identifying the taste buds, as the AT antibody labelled microtubules in cytoplasm, cilia and axons and taste buds were identified as several AT-IR cells clustered, forming a cup or pear shape structure in the pharyngeal epithelium. These taste bud cells were ciliated at the apical surface. Nerve fibers were located at the basal side surface of the taste bud. The relative size of these taste buds in the different pharyngeal regions was evaluated in serial sections by counting the number of AT-IR taste cells within taste buds in a through focus analysis of the labelled tissue sections. Taste cells within a taste bud were counted if a nucleus was clearly seen and if AT-IR supra nuclear cytoplasm extended to the epithelial surface. Ten lampreys were initially selected for this study. Due to technical limitations, quantitative taste bud data from seven lampreys and quantitative AT-IR cells per taste bud data from three of the seven lampreys were utilized. The number of taste buds in different regions of the pharynx and the number of AT-IR taste cells per taste bud was recorded and presented as mean ± standard error (SE). A Friedman Test followed by a post-hoc Dunn-Bonferroni test was conducted to assess the significance of differences in taste bud distribution and number of taste cells per taste bud at different regions of the pharynx.

### Electrophysiological recordings

Pre-feeding juvenile sea lampreys (Evans et al. 2021) (n = 48, 135 mm to 178 mm; 1.6–3.3 g) and parasitic juveniles (n = 7, 150–490 mm; 16.3–401 g) were examined for this study. Each was anesthetized using dechlorinated water solutions of 0.5 g/L of tricaine methane sulphonate (MS-222). The pharynx was isolated by a transverse cut rostral to the first external gill pore and caudal to the seventh external gill pore. The isolated pharynx was then placed in cold (4–8°C) Ringer’s solution. The dorsal surface was pinned down and a shallow incision was made on the ventral surface along the ventral midline. The stomach was located and carefully excised to expose the ventral side of the pharyngeal cavity. The ventral surface of the pharynx was cut through the ventral midline, exposing the inner pharyngeal surface containing the taste buds. Injections of 2 mg/kg of body weight, of gallamine triethiodide solution was applied to several locations on the pharynx to inhibit muscle contractions. The preparation was transferred to the electrophysiological rig and pinned down with the dorsal side face down and the pharyngeal cavity exposed. While the location of the taste buds was known from the immunochemical experiments;2% methylene blue supravital staining of the taste receptor cells was initially used on these preparations to visually guide the positioning of the recording electrode onto the taste buds.

Electrophysiological methods were utilized to record taste cell action potentials by placing the recording electrode above taste buds (Avenet and Lindemann 1991; Cummings et al. 1993; Gilbertson et al. 1992). A micromanipulator was used to place a glass electrode with a tip diameter between 50–250 μm external to the surface over the location of taste buds that were visible with methylene blue supravital staining (Fig. 3a). To start, one of the six pharyngeal regions was randomly selected, and the recording electrode was placed in the center, halfway between the two inner gill slits and moved slowly until spontaneous activity was observed. After the series of tastant solutions were applied for 5 s to this location, the electrode was moved to a different region and the applications of the tastants were repeated. All recordings were obtained from the right side of the lamprey’s pharynx. Consecutive test solution deliveries were at least 2 min apart to avoid adaptation. At each location, each tastant was delivered at least 3 times to elicit three separate responses and each tastant solution delivery was 5 s in duration. The order of the regions recorded from were randomized and the tastant solutions were applied in random order. From a single animal, 2–4 different regions were tested with a set of 3 repetitions of at least 3 compounds, as well as with Ringers solution (blanks), before the prep tissue stopped responding. Ringers’ solution (blank) was tested in between switching from one tastant test to another to monitor for potential mechanosensory responses and to wash out potential residue of the tastant solutions. The signals were amplified using an A-M systems differential amplifier model 1800 and an AD instruments Powerlab 4/30 was used to convert the analog data to digital. The data were displayed on Labchart 6.0 software.

A 9-chamber stimulant delivery/cooling system was utilized to chill and deliver background medium (Ringer’s solution) and the tastant solutions over the pharynx at the same temperature as the chilled preparation (Green et al. 2017). Briefly, the pharynx was dissected out of the body since the dissected pharynx, with various interstitial cells exposed, was used for these recordings, the tissue was immersed in Ringer’s solution to prevent hypo-osmotic effects. Ringer’s solution was continuously perfused over the pharynx via a gravity fed, valve-controlled test solution delivery system and an electronically triggered, computer-controlled, three-way solenoid valve. This allowed for fast switching between background Ringer’s solution and a desired test solution with no interruption in flow to the pharyngeal surface. Some experiments required two or three washes with Ringer’s from the stimulus delivery apparatus solutions before no effect on spike rate was observed.

### Tastant test solutions

Tastants were dissolved in ice-cold lamprey Ringer’s solution (130 mM (millimoles)/L NaCl, 2.1 mM/L KCl, 2.6 mM/L CaCl_2_, 1.8 mM/L MgCl_2_, 4 mM/L Hepes, 4 mM/L dextrose, 1 mM/L NaHCO_3_) and pH was monitored and adjusted using 2 M/L NaOH to 7.3–7.4. Two larval brook lamprey tastants (alanine, and sucrose) (Baatrup 1985), a sea lamprey solitary chemosensory cell stimulant (glycine) and bile acid taurodeoxycholic acid (TDCA) (Daghfous et al. 2020) were applied over each of the pharyngeal regions. Glycine stimulation indicates a possible solitary chemosensory cell response (Daghfous et al. 2020). Bile acids are potent solitary chemosensory cell stimulants in lampreys (Daghfous et al. 2020), and are also tastants for teleosts such as rainbow trout (Yamashita et al. 2006) and channel catfish (Rolen and Caprio 2008). The concentrations used were 10 mM for all tastants, except for TDCA, which was applied at 1 mM. These concentrations were the minimal concentrations that reliably caused increases in spike rate. ATP is a tastant that activates feeding in blood-sucking mosquitoes (Galun et al. 1963; Jové et al. 2020). As sea lampreys are also blood feeders, 0.2 mM ATP was prepared in Ringer’s solution and applied at a constant flow rate over a 5-s period.

### Spatial comparison of chemosensory responses

Electrophysiological recordings were analyzed using Labchart8 software from ADinstruments (Sydney, Australia). Spikes were filtered using a detection threshold of 5 SD (standard deviation) above baseline voltage. SD was calculated using a 1 min interval before stimulus onset, since this allowed for a stable representation of the baseline voltage. A spike histogram of the recording trace was produced using the spikes that reached detection threshold. Using the spike rate histogram, the highest spike rate count in a five second bin, up to 15 s after stimulus application, was subtracted by the spontaneous spike rate one minute before stimulus application. A five second bin was used to obtain the response spike rate (spikes/second) as the length of stimulus application was five seconds. A time window of 15 s following stimulus application was allowed for in which the response spike rate was calculated due to variability in the delay of response onset between different preparations. Additionally, to control for bias towards random fluctuations in spike rate we calculated the peak pre-stimulus spike activity in a 5-s bin during the one minute of spontaneous activity and compared this value to the response rate. IBM SPSS software was used to perform a mixed linear model analysis with restricted maximum likelihood, to compare spontaneous rate, response rate and peak pre-stimulus rates, where the regions and the time (response, spontaneous, and peak pre-stimulus activity) were fixed factors, and the animal ID was a random factor. In all preparations, differences were considered significant at the *p* < 0.05. Mean and standard deviation values are expressed in spikes/sec. For each test, the tastant was applied three times. The spike rate for the three trials at each recording, as well as the preceding spontaneous spike rate were averaged to yield a value of mean spikes/sec per animal for each region. These values of spike rate per second per animal for each tastant was then averaged across all animals that were tested in that specific region. The response column designates the spike rate (spikes per second) from a 5-s bin following a five second application of the tastant. The spontaneous spike rate was calculated as the average spike rate during the one minute immediately preceding the tastant application.

## Results

### Distribution of pharyngeal taste buds

**Fig. 2:**
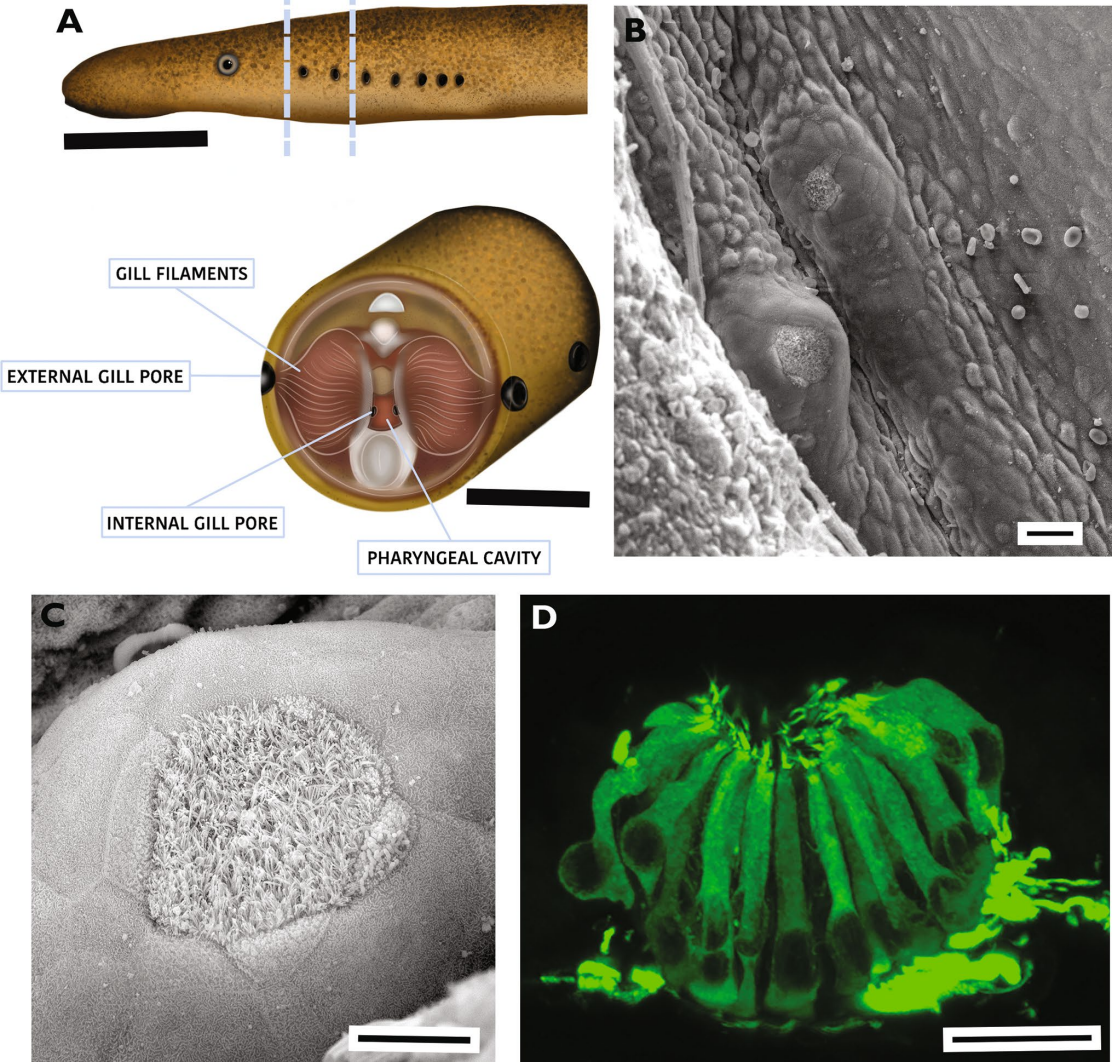
Sea lamprey pharyngeal taste buds in the pre-feeding juvenile sea lamprey. **A** Drawing of the pre-feeding juvenile phase sea lamprey with seven external gill pores at the outer skin surface and a cross-sectional view of the location of the pharyngeal cavity. The internal gill pores connect the pharyngeal cavity to the gills. The top scale bar is 10 mm; the bottom scare bar is 5 mm. **B** A scanning electron microscope view of the pharyngeal surface shows two papillae with taste buds on the lateral pharyngeal wall in a newly transformed juvenile. Bar is 25 μm. **C** A scanning electron microscope view of the ciliated surface of a taste bud located on a pharyngeal papilla. Bar is 10 μm. **D** A confocal image of a pharyngeal taste bud immunolabeled with anti-acetylated tubulin shows apical cilia, fusiform shaped taste cells. Axons and axon fascicles are located at the basal surface of the taste bud. Bar is 10 μm

**Fig. 3:**
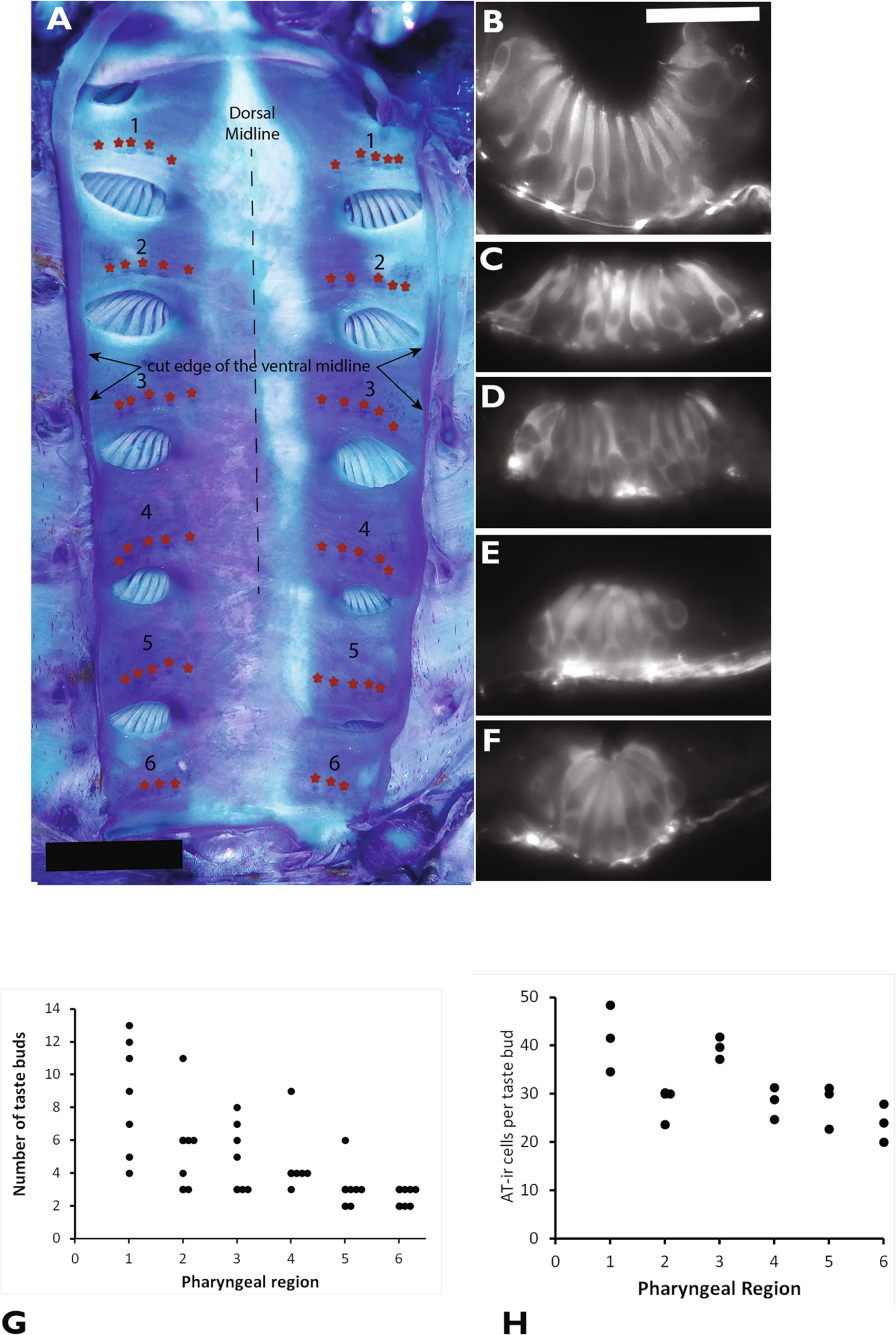
Taste buds on the lateral surface of the pharyngeal cavity in the pre-feeding juvenile stage sea lamprey **A**. A stereomicrograph of the pharyngeal cavity cut along the ventral midline, opened to expose the seven lateral internal gill pores located at the brancheal arches (1–7), then stained with 2% methylene blue. Small asterisks were added above presumptive taste buds that stained with the methylene blue. The scale bar is 1 mm. **B**–**F** are examples of taste buds labeled with AT immunocytochemistry. The scale bar shown in **B** is 25 μm and applies for **B**–**F**. **B** is located in lateral pharyngeal region 2. **C** is located in lateral pharyngeal region 3. **D** is located in lateral pharyngeal region 4. **E** is located in lateral pharyngeal region 5. **F** is located in lateral pharyngeal region 6 **G**. The number of taste buds in the six pharyngeal regions between the seven internal gill pores (n = 7 lampreys, length of approx. 14 cm, weight 2.4 to 2.6 gm). According to the Friedman Test followed by a post-hoc Dunn-Bonferroni test, the number of taste buds in Region 1 is significantly higher compared to Region 5 and Region 6 (p < 0.05) **H**. The number of AT-IR cells located in taste buds at the six lateral pharyngeal locations between internal gill pores. According to the Friedman Test followed by the post-hoc Dunn-Bonferroni test, the number of AT-IR cells per taste bud in Region 1 and in Region 3 is significantly higher compared to Region 6 (p < 0.05). No significant difference is found in the number of AT-IR cells in Region 2, Region 4 and Region 5. Only AT-IR cells with a clearly visible nucleus were included in these counts

In the pre-feeding juvenile, prominent papillae were located on the lateral surface of the pharyngeal cavity between the internal gill pores (Fig. 2a, b). The apical surface of these papillae was ciliated and easily distinguished from the surrounding flat-surfaced epithelium (Fig. 2c). Signs of solitary chemosensory cells, which are microvillar (Suntres et al. 2020) were not seen on the surface of the epithelium close to the taste buds, rather the micro-ridges characteristic of epithelial cells was apparent on the epithelium surrounding the cilia extending from the taste buds (Fig. 2c). These ciliated taste bud cells were also prominent in anti-AT labelled preparations viewed by confocal microscopy, and axons were located adjacent to the basal surface of these cells (Fig. 2d). The locations with pharyngeal taste buds between the six internal gill pores (Region 1 to 6) were recognizable in whole mount preparations of the pharynx stained with methylene blue (Fig. 3a). These six lateral inter-gill pore locations contained taste buds with ciliated AT-ir neuro-epithelial cells and axons extending along the basal surface of the taste buds (Fig. 3 b–f).

In the 7 specimens that were examined, the total number of pharyngeal taste buds ranged from 40 to 96 taste buds and the average number of taste buds was 62 pharyngeal taste buds per lamprey. The number of taste buds in each of these 6 pharyngeal regions varied between 2 and 13 taste buds per region (Fig. 3g). There was a trend of more taste buds located in rostral pharyngeal regions. The Friedman test followed by post hoc Dunn-Bonferroni analysis (N = 7 lampreys; df = 5; H (5) = 20.1; p < 0.001) indicated significantly more taste buds in Region 1 (9 ± 1 taste buds) than in Region 5 (3 ± 1 taste buds, adj. P-value: 0.005) and Region 6 (mean = 3 ± 1 taste buds, adj. P-value: 0.001). The taste bud counts were similar in Region 2 (mean = 6 ± 1 taste buds), Region 3 (mean = 5 ± 1 taste buds) and Region 4 (mean = 5 ± 1 taste buds). The number of taste buds in Regions 2, 3 and 4 was not significantly greater than the number of taste buds in Regions 5 and 6, with p-values greater than 0.05. However, overall, Region 1 contained more taste buds than Regions 5 and 6, whereas Regions 2, 3 and 4 contained a similar number of taste buds.

The number of AT-ir cells per taste bud was compared in the six pharyngeal regions, as an indicator of taste bud size (Fig. 3h). The number of AT-IR taste cells in a single taste bud varied from 15 to 68 AT-IR cells per taste bud. The Friedman test, followed by post hoc Dunn-Bonferroni analysis, were conducted on a sample size of 3 lampreys with degrees of freedom (df) equal to 5, yielding a critical value H (5) of 12.24. The posthoc Dunn's test using an alpha of 0.05 indicated that the number of AT-IR cells per taste bud in Region 1 (mean = 42 ± 8 AT-IR cells) and in Region 3 (mean = 40 ± 6 AT-IR cells) was significantly higher than the number of taste cells per taste bud in Region 6 (mean = 26 ± 5 AT-IR cells) (p = 0.032). The number of AT-IR cells per taste bud in Region 2 (mean = 26 ± 6 AT-IR cells), Region 4 (mean = 29 ± 7 AT-IR cells) and in Region 5 (mean = 28 ± 5 AT-IR cells) was not statistically different from the number of AT-IR cells in Regions 1, 3 or 6. Overall, pharyngeal taste buds were more abundant in Region 1 than in Regions 5 and 6, and the taste buds were larger in Region 1 compared to Region 6.

### Pharyngeal chemosensory taste receptor cell responses to canonical tastants and to ATP

**Fig. 4:**
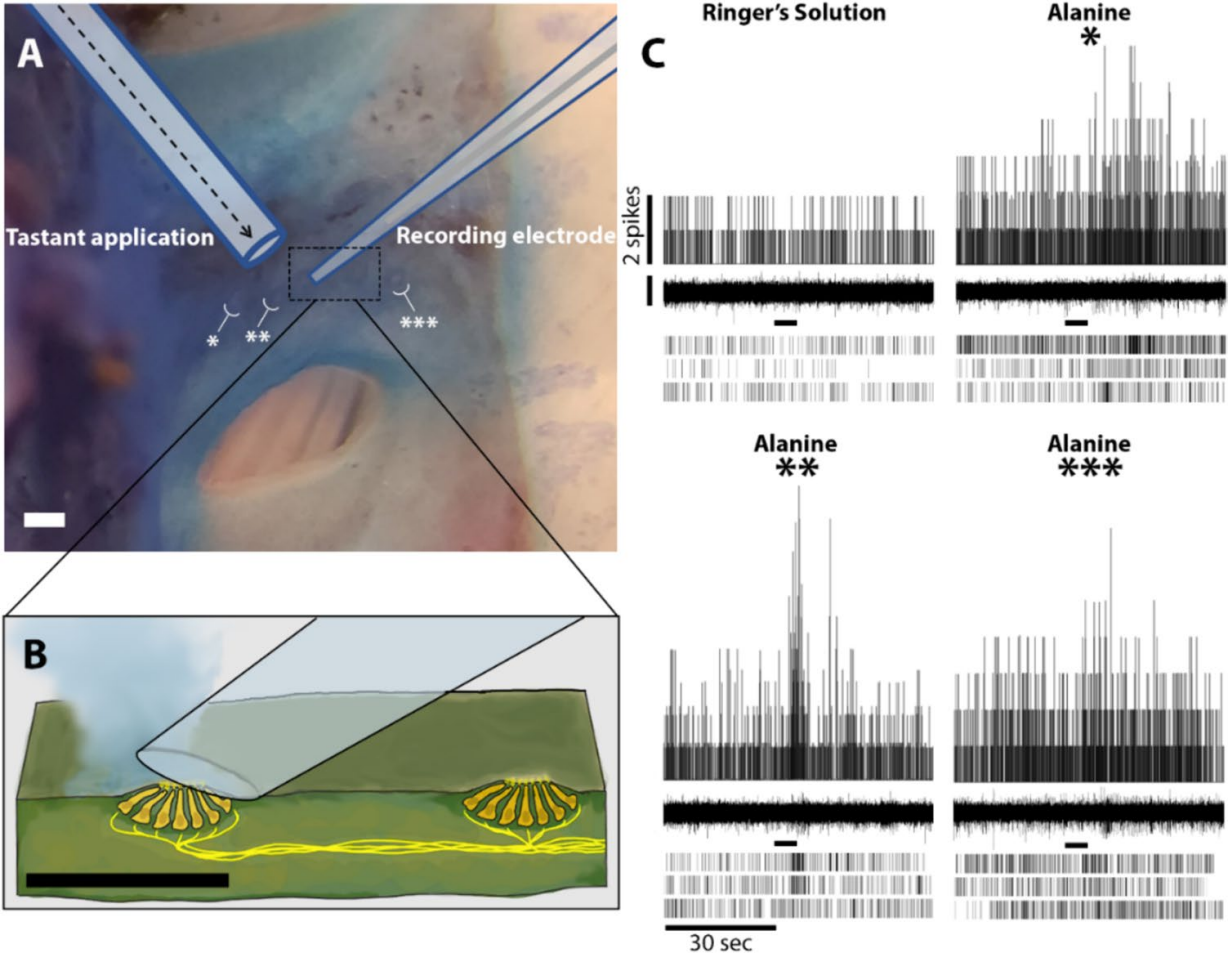
Taste receptor cell activity recordings from the lateral surface of the sea lamprey pharynx. **A** Stereomicrograph of the experimental setup for recording chemosensory responses from taste buds in the pharyngeal cavity. Medial is on the right. Methylene blue (2%) was supravitally applied. The recording electrode is positioned above the taste bud. Other locations with taste buds are at locations beside asterisks (*, ** and ***). Scale bar is 200 µm. **B** The diagrammatic illustration of recording preparation shows the location of the taste buds, the tastant application (blue-gray shading) and recording electrode on the lateral region of the pharynx. Scale bar is 200 µm. **C** Taste cell activity recorded from 3 separate taste buds indicated as *, ** and *** during the application of L-alanine for 5 s. The single frequency histogram was derived by overlaying the activity from the three single raster plots

**Fig. 5:**
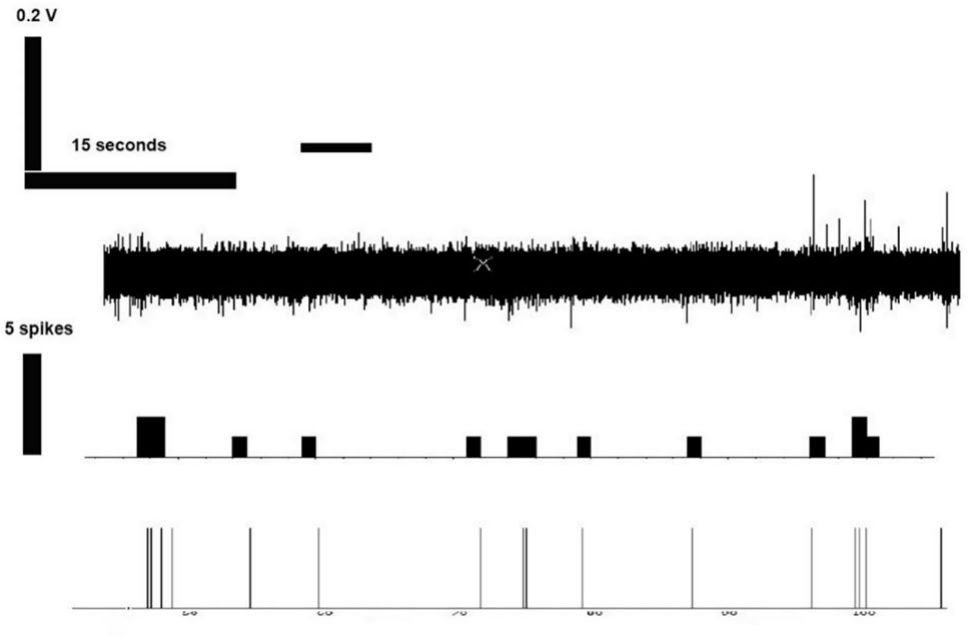
Spike activity is not associated with the application of l-alanine to the most rostral region of the pharyngeal cavity. This area does not contain taste buds. l-alanine was applied for 5 s. The top tracing shows the raster, the second line shows a frequency histogram

**Fig. 6:**
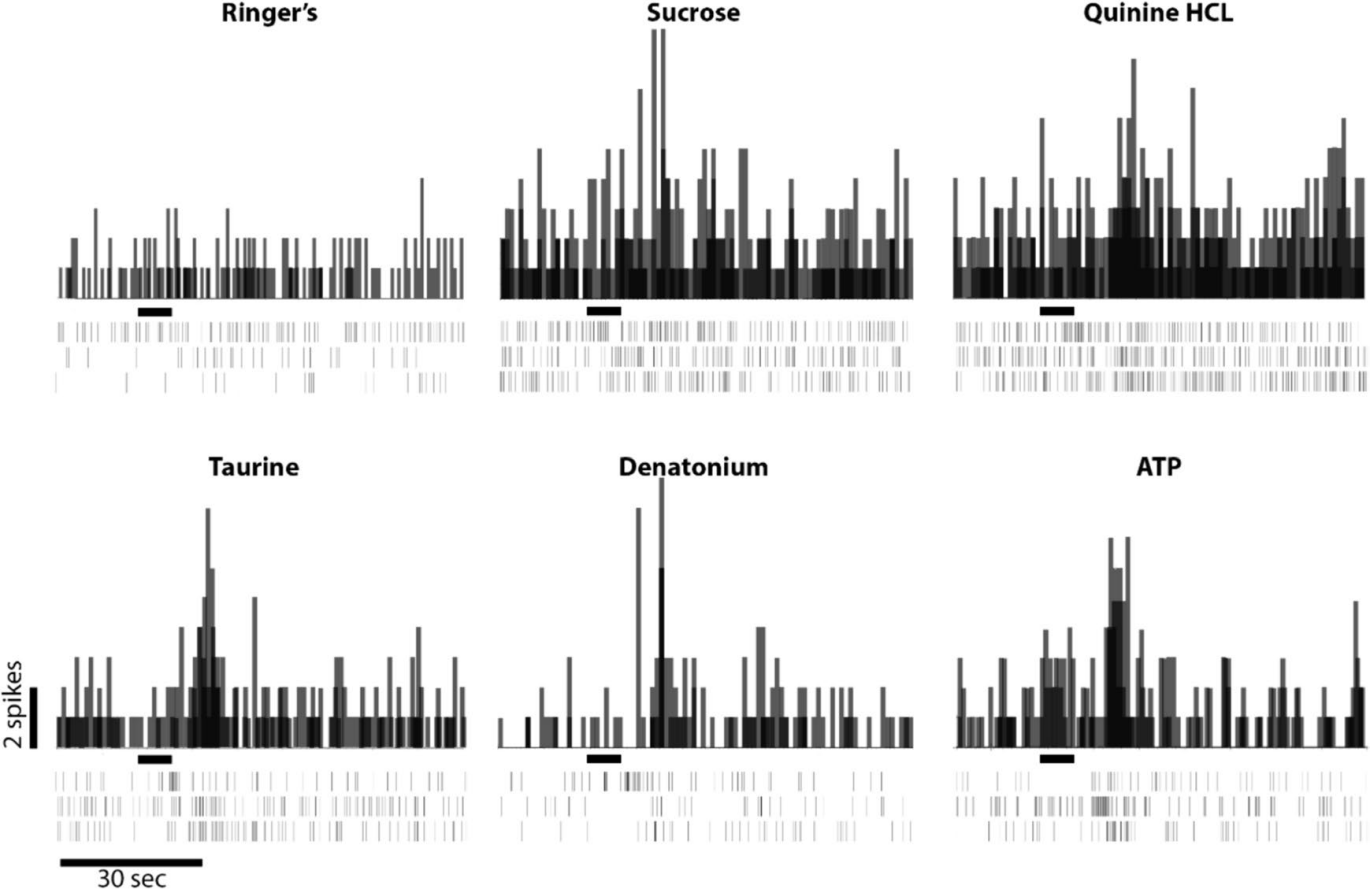
Taste bud chemosensory responses to Ringer’s wash (blank), 10 mM sucrose, 10 mM quinine HCL, 10 mM Taurine, 10 mM denatonium, and 200 µM ATP are shown here. The frequency histograms and raster plots show 25 s of pre-stimulus activity, a 5 s stimulus application and 60 s of post-stimulus activity. The onset of the tastant test solution application and duration are indicated by black horizontal bars between each pair of plots. The single frequency histogram was derived by overlaying the activity from the three single raster plots

**Fig. 7:**
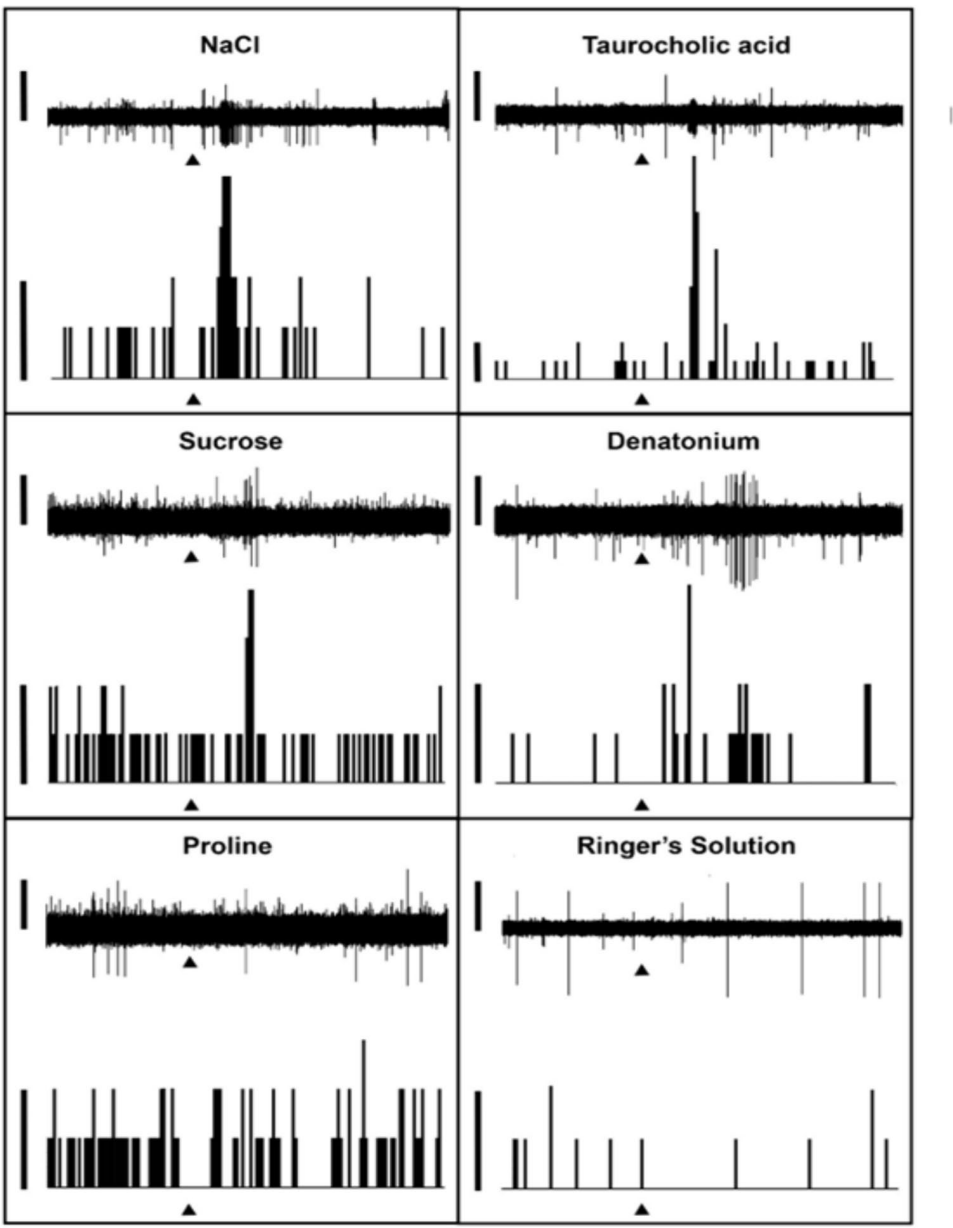
Taste bud chemosensory responses in the parasitic juvenile stage of the sea lamprey. The raster plot is shown at the top and the frequency histogram is shown at the bottom. Neural responses show increased receptor cell activity in response to 1 mM NaCl, taurocholic acid, sucrose, and denatonium, while 1 mM proline did not elicit a similar response (substance application onset indicated by arrow; receptor cell activity trace scale bar: 200 mV). Spike histogram analysis of these responses show the number of action potential spikes recorded within 0.5 s bins (substance application onset indicated by arrow; scale bar: 2 spikes)

Chemosensory neural responses were tested in the pharyngeal regions containing taste buds, as determined from assessments of AT-immunolabeled tissue and from the location of presumptive taste buds, seen in tissue supravitally stained with methylene blue (Fig. 3). The tastant test solutions were delivered for five seconds through a micropipette positioned adjacent to the taste buds and a recording electrode was positioned over the taste buds (Fig. 4a, b). Activity representing action potentials was observed at these pharyngeal inter gill pore regions and spike responses to chemical stimulation were seen clearly from these pharyngeal territories containing taste buds (Fig. 4c). Tastants that were tested included the amino acids l-arginine and l-alanine, aversive taste stimuli mediated by quinine and sweet taste by sucrose (Fig. 5). Receptor cell activity responses to sucrose and alanine, as previously seen in the pharynx of larval brook lampreys (Baatrup 1985), further affirmed that the electrode was recording gustatory responses. These chemosensory responses were not observed when the recording electrode was positioned rostral to the first gill (Fig. 5) a region which is devoid of taste buds. Compounds not previously tested on the lamprey pharynx: denatonium, ATP, and the bile acid TDCA were also stimulatory. Both 0.2 mM and 1 mM ATP elicited receptor cell activity responses in 6 out of 9 preparations. The bitterants denatonium and quinine were also stimulatory. The denatonium applications stimulated robust responses in 4 out of 7 preparations. Other amino acids (l-proline, l-arginine) and the bile acid derivatives (taurine, cholic acid) also elicited robust responses. These responses had an initial burst where activity peaked immediately, decreased slightly, then plateaued for 5–10 s, while still staying above baseline activity (e.g. Fig. 6). Taste receptor responses were also tested from parasitic juvenile lampreys. These responded to the same tastants as the pre-feeding juvenile, including NaCl, taurocholic acid, sucrose and denatonium, but not proline (Fig. 7), which also did not stimulate responses from larval brook lamprey (Baatrup 1985).

### Regional comparison of pharyngeal chemosensory receptor cell activity responses

**Fig. 8:**
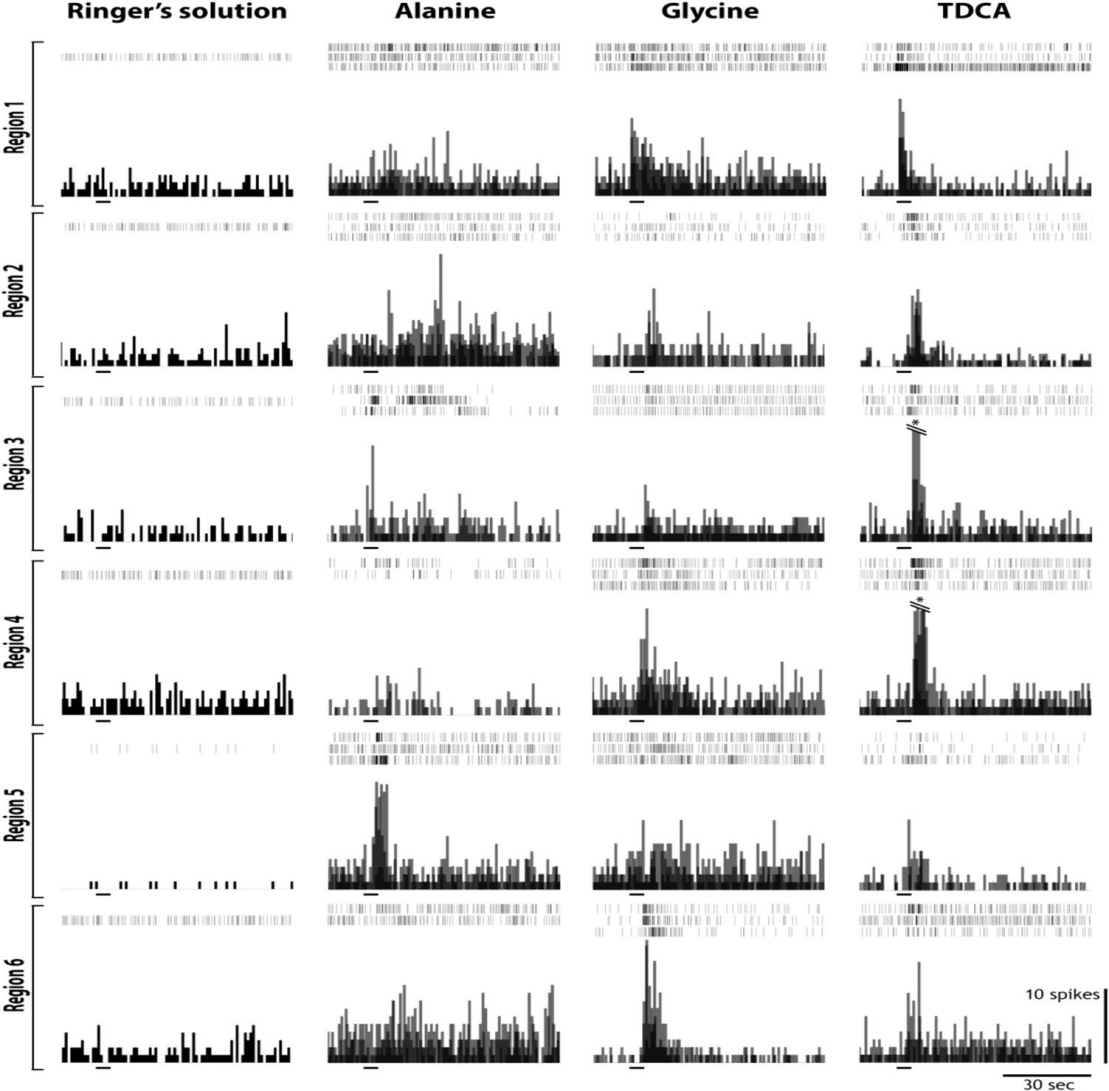
Sample spike histograms and their associated raster plots for three trials per recording for each compound tested from the internal wall of the pharyngeal cavity, between the 6 gill pores. Region 1 is located between gill pore 1 and gill pore 2. located caudal to a gill pore. Region 1 is caudal to gill pore 1; region 2 is located caudal to gill pore 3 etc. The raster plots and the histograms are aligned and show baseline activity for 15 s prior to the test, then 60 s following the 5 s test application. The single frequency histogram was derived by overlaying the activity from the three single raster plots. The black horizontal bar indicates the onset and duration of the stimulus application

**Fig. 9:**
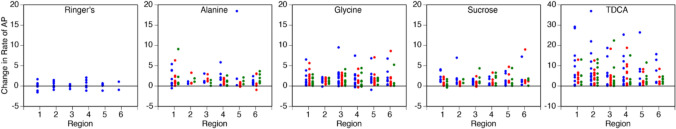
Changes in the rate of action potentials (AP, spikes/s): Graphs visualize change in firing rate for each tastant tested in the six pharyngeal regions. These values were obtained by subtracting the response rate from the baseline spike rate. Increases in firing rate were observed for all tastants in all regions. Values are from 30 separate animals. Blue is the first application; red is the second application and green is the third application. 10 mM l-Alanine, glycine and sucrose were applied and 1 mM TDCA was applied. Taste receptor cell activity activity following the application of each compound showed statistically significant stronger response magnitudes than to Ringer’s application

The regionality of chemosensory responses to the tastants glycine, alanine, sucrose and TDCA was investigated at the six pharyngeal locations containing taste buds in the pre-feeding juveniles (see Fig. 2). Taste receptor cell responses were recorded from forty-six (n = 46) pre-feeding juvenile sea lampreys and were subjected to statistical analysis of the regionality of pharyngeal chemosensory receptor cell activity responses (Fig. 8, Table 1). Of these 46 pharyngeal preparations, 33 were analyzed for responses to 1 mM TDCA, 32 preparations for 10 mM glycine, 27 preparations for alanine, and 24 preparations for sucrose application. A mixed linear model analysis was conducted to determine statistically significant effects on spike rate between different regions. Significant differences were found for all the tastants when comparing the response spike rate to the spontaneous activity or to the peak pre-stimulus activity. The spike rate increases due to TDCA application compared to spontaneous activity (n = 33, (f = 59.3) p < 0.001), and peak pre-stimulus activity (n = 33, (f = 43.6) p < 0.001) were significant. Spike rate increases due to alanine application compared to spontaneous activity (n = 27, (f = 47), p < 0.001) and peak pre-stimulus activity (n = 27, (f = 12.2), p < 0.001) were also significant. Effect on spike rate due to glycine application was also significant compared to spontaneous activity (n = 32, (f = 29.6), p < 0.001) and peak pre-stimulus activity (n = 32, (f = 6.9), p < 0.01). From sucrose application the difference between response spike rate and spontaneous activity (n = 24 (f = 16.5), p < 0.01), and peak pre-stimulus activity (n = 24, (f = 4.7), p < 0.05) was also significant. For application of Ringers’ there was also a significant effect on spike rate when comparing between the response rate and the spontaneous activity (n = 34, (f = 23.5), p < 0.01), however when comparing the response rate to the peak pre-stimulus activity the difference was no longer significant (n = 34, (f = 0.665), p = 0.517). These results indicate that the applications of these tastants have a significant effect on increasing the spike rate in the pharynx which cannot be attributed to random fluctuations in spontaneous receptor cell activity. Between regions there were no significant differences on spike rate in response to TDCA (p = 0.169), glycine (p = 0.268), and sucrose (p = 0.491), however alanine did show a significant effect on spike rate based on the region (p < 0.01). Pairwise comparisons indicate that this difference was driven by differences in spike rate between region one and region four (Fig. 9). Overall, these findings suggest that responses to these tastants are present in each of the regions (Fig. 9).

**Table 1:** Summary of mean spike rate for each tastant that was tested from taste buds in the six pharyngeal across all animals. For each test, the tastant was applied three times

Region ​	Alanine		Glycine		TDCA		Sucrose	
	Response Mean spikes/sec ± SD N​	Spontaneous Mean spikes/sec ± SD N​	Response Mean spikes/second ± SD N​	Spontaneous Mean spikes/sec ± SD N​	Response Mean spikes /sec ± SD N​	Spontaneous Mean spikes /sec ± SD N​	Response Mean spikes/sec ± SD N​	Spontaneous Mean spikes/sec ± SD N
1​	4.13 ± 3.0 8​	1.86 ± 1.65 8​	2.87 ± 2.62 11​	1.71 ± 2.44 11​	9.5 ± 9.05 11​	1.72 ± 1.96 11​	2.475 ± 2.19 11​	1.35 ± 1.45 11​
2​	2.796 ± 1.92 8​	1.42 ± 0.90 8​	2.29 ± 0.97 12​	1.28 ± 0.86 12​	8.29 ± 6.06 12​	1.12 ± 0.87 12​	3.11 ± 2.81 9​	1.21 ± 1.07 9​
3​	2.88 ± 1.50 7​	1.22 ± 1.26 7​	3.05 ± 2.10 10​	1.12 ± 1.09 10​	10.54 ± 9.52 9​	1.81 ± 1.44 9​	2.04 ± 1.3 9​	1.47 ± 1.11 7​
4​	2.56 ± 1.50 9​	1.33 ± 1.33 9​	3.75 ± 1.95 9​	1.70 ± 1.14 8​	6.53 ± 6.63 13​	1.42 ± 0.92 13​	3.98 ± 4.58 8​	1.28 ± 0.94 8​
5​	3.62 ± 3.72 4​	1.54 ± 1.42 4​	3.67 ± 2.28 9​	1.68 ± 1.41 9​	5.63 ± 5.47 8​	1.60 ± 1.5 7​	2.75 ± 2.19 7​	1.37 ± 1.31 7​
6​	3.59 ± 2.26 7​	2.17 ± 2.16 7​	2.60 ± 2.51 8​	1.11 ± 0.96 7​	4.85 ± 3.33 9​	1.41 ± 1.20 9​	3.88 ± 5.59 6​	1.76 ± 2.69 6​

The spike rate for the three trials at each recording, as well as the preceding spontaneous spike rate were averaged to yield a value of mean spikes/sec per animal for each region. These values of spike rate per second per animal for each tastant was then averaged across all animals that were tested in that specific region. The response column designates the spike rate (spikes per second) from a 5-s bin following a five second application of the tastant. The spontaneous spike rate was calculated as the average spike rate during the one minute immediately preceding the tastant application.

SD is standard deviation. N is the number of specimens

## Discussion

This study shows abundant taste buds and chemosensory responses at six pharyngeal locations during the pre-feeding juvenile stage of the sea lamprey, immediately prior to feeding on the blood and tissues of host organisms, as well as during the parasitic juvenile stage. These findings support the idea that gustation is important during the juvenile stage, when sea lampreys grow rapidly and acquire essential nourishments such as fatty acids, amino acids and carbohydrates by consuming blood and fluids of prey (Farmer 1980; Kleerekoper and Mogensen 1963; Youson 1981). Since the oral feeding apparatus was also toothed and suctorial in early lampreys during the Paleozoic era (Miyashita et al. 2021; Wu et al. 2023), the gustatory chemosensory system may have also been important during early lamprey evolution.

### Taste buds in the lamprey pharynx

SEM examination showed that the taste bud cells in pre-feeding juvenile sea lamprey contain apical projections and are elevated above the surrounding epithelium, as previously observed in the pharynx of larval brook lamprey (Baatrup 1983a). All six lateral pharyngeal locations contained ciliated taste buds, when viewed by acetylated tubulin immunocytochemistry, previously used to label the taste buds in sea lamprey larvae (Barreiro-Iglesias et al. 2008). At each of these locations, the taste cells were organized into taste buds, as in other vertebrate species (Murray et al. 1969; Farbman and Yonkers 1971; Yoshie et al. 1990). Northcutt (2004) observed that primitively, taste buds are restricted to the oropharynx of vertebrates, as this location for taste buds is seen in lampreys (Baatrup 1985), cartilaginous fish (e.g. Whitear and Moate 1994), primitive ray-finned fish (e.g. Norris 1925) and tetrapods (e.g. Ganchrow and Ganchrow 1985; Miller and Smith 1984; Northcutt et al. 2000). Teleost fish usually have taste buds on the lip, mouth, oropharynx and on the skin covering the body (Atema 1971; Fishelson et al. 2004). Taste buds have been found in the pharynx, specifically on the gill arches of mullets (*Mugal cephalus*), killfish (*Fundus heteroclitus*) (Hossler and Merchant 1983), channel catfish (Atema 1971) and carp (Hansen et al. 2014). Solitary chemosensory cells, located on the exterior surface of the gill pores in the spawning phase sea lamprey responded to bile acids and the amino acids glycine, proline and glutamate, but not arginine (Daghfous et al. 2020),

In post-metamorphic lampreys, including sea lampreys, two passageways extend caudally from the buccal cavity-one leading to the esophagus and the second to the pharynx (Dawson 1905; Hardisty 1979; Manzon et al. 2015; Evans et al. 2021). The lamprey’s toothed sucker-like oral apparatus maintains attachment on prey and fluid entering the mouth is pumped through the pharynx and out the gill pores (Kawasaki and Rovainin 1988) allowing for flow over taste buds that are located on the lateral surface of the pharynx, thus enabling tasting while the lampreys’ sucker is attached to the fish. The pharyngeal location of taste buds also allows for tasting with respiratory ventilation of water into and out of the pharynx through the gills. Since the pharyngeal taste buds are exposed to these sources of water, chemosensory detection by the taste buds may be involved in rejecting or accepting the food based on the chemicals that contact the taste buds. Aquatic vertebrates such as teleost fish utilize the taste system to search for food and control the level of food consumption (Michel 2006; Kasumyan et al. 2009). Taste buds on the pharyngeal arches may be checking the water quality and detecting the food-related chemicals to elicit quick consumption, rejection or to control the rate of food consumption (Hara et al. 1994; Kiyohara et al. 1980; Linser et al. 1998; Northcutt et al. 2000).

In this study, taste receptor cell activity was recorded from six rostro-caudal pharyngeal regions that contained taste buds. Solitary chemosensory cells may also be present in the pharynx, as these cells are located on the outer surface of the gills, largely during the spawning phase lampreys (Daghfous et al. 2020; Suntres et al. 2020). In this study of the pharynx, more taste buds and larger taste buds were in the most rostral location compared to the most caudal location, however taste receptor cell activity responses in all regions to sweet, amino acid and bile acid tastants were similar with respect to the frequency of action potentials. Currently, the identity of the cranial nerves innervating these taste buds in sea lampreys is unreported. However, Alcock (1898) described respiratory motor branches of the glossopharyngeal nerve in the approximate vicinity of the first two external branchial pores, while the vagal nerve branches were located above the third to seventh branchial pores. The lack of tastant regionality observed in the current study of the pharynx may be associated with the different motor pathways that each cranial nerve activates. Each cranial nerve would still need to be able to detect each of the stimulants but would regulate different motor outputs. For example, in catfish, removal of the vagal lobe prevented swallowing of food, but the fish still maintained the ability to capture food into their mouths (Atema 1971). On the barbels of catfish, taste buds are innervated by the facial nerve, which functions for prey search and capture (i.e. to locate and intake food into the mouth) while taste buds located in the posterior region of the oral cavity and the gill arches are associated with the vagal nerve and provide tastant detection for swallowing or expelling food inside the mouth (Finger 2009).

### Distribution and size of pharyngeal taste buds

In this study, there were more taste buds and larger taste buds in the most rostral pharyngeal region (Region 1) compared to the most caudal region (Region 6). In another basal vertebrate, the hagfish, a higher number of Schreiner organs structures, which exhibit structural similarities to taste buds, were also located in the rostral region of the pharynx compared to the tail end of the pharynx (Braun 1998; Northcutt 2004). The rostral part of the lamprey pharynx, which has a higher number of taste buds, may be involved in the quick foraging of food and quick rejection of unpalatable food by the sea lamprey. Interestingly, the tongue, an organ with taste buds in jawed vertebrates, evolved because of changes to the most rostral branchial arch (reviewed by Pennisi 2023).

In some teleost fish, the posterior part of the oropharyngeal cavity tends to have a higher concentration of taste buds than the anterior region (Fishelson et al. 2012; Kiyohara et al. 1980). However, in the minnow, the anterior part of the gill rakers exhibits a higher density of taste buds than the posterior region (Kiyohara et al. 1980). This difference in distribution patterns in teleosts is attributed to the posterior region's role in serving as a boundary between the esophagus and the mouth, allowing for the final assessment of food before swallowing.

The solitary chemosensory cells may also have a role in chemosensory input during feeding. These are located on the outer surface of the gills in pre-feeding juveniles and in parasitic juvenile sea lampreys (Suntres et al. 2020) and may monitor food quality (Daghfous et al. 2020), as some nitrogenous compounds stimulate aversive behavioural responses (Dissanayake et al. 2019).

## Taste responses

### Tastants

This is the first study to report chemosensory receptor cell activity responses from the pharynx of sea lamprey, and the first study of gustation in any lamprey following metamorphosis and during feeding on the blood of prey. Chemosensory responses to canonical tastants such as sweet (sucrose), bitter (denatonium) and the amino acids alanine and glycine, support the function of taste cells in the six lateral pharyngeal regions. While sucrose was tested as an indicator of sweetness, future study should test for responses to sweet molecules such as glucose, since these lampreys feed on the blood of the prey. As glucose is a component of the lamprey Ringer’s solution, the chemical composition of the Ringers solution should be considered for these future experiments.

To investigate whether specific tastants only stimulate specific regions of the pharynx, we tested a set of four compounds representing amino acids (alanine, glycine), sweet (sucrose), bile acids (TDCA) in each pharyngeal region while measuring the receptor cell activity of the taste buds in these regions. All four compounds elicited significantly higher spike rates compared to spontaneous activity. The bile acid TDCA showed the greatest mean response magnitudes. The amino acids l-glycine and l-alanine, as well as sucrose also stimulated statistically different response magnitudes compared to the Ringers’ application but with lower mean response magnitudes than TDCA. The mixed linear model revealed no statistically significant interaction between the regions on spike rate for TDCA, glycine or sucrose, and Ringers, however there was a significant difference for alanine, between regions one and four. These findings suggest that there is no apparent chemosensory regionality in the pharynx of the sea lamprey for TDCA, glycine or sucrose. Caprio et al. (2015) observed two types of taste fibers, one responding strongly to l-proline and betaine and the other responding more strongly to l-alanine and glycine in the barbels of the Japanese sea catfish (*Plotosus japonicus*). Similarly, our findings indicate that taste cells in region four may lack receptors for alanine or the taste cells in this region are less sensitive to certain amino acids. There may be inter-individual chemosensory differences between the lampreys, since in some preparations, responses to alanine, sucrose, and glycine were observed in a single region, while in other lampreys there were no responses to alanine, glycine, or sucrose in the same pharyngeal region.

These results reaffirm the notion that the gustatory system of a basal vertebrate relies on the detection of basic constituents of organic matter, such as sugars, amino acids, and alkaloids, underlying the importance and conserved functions of the gustatory system. ATP, a molecule used for short term energy at the cellular level, consistently stimulated pharyngeal responses in the sea lamprey. In mosquitoes, ATP in feeding solutions is a phagostimulant for blood sucking, including engorgement (Galun et al. 1963; Jové et al. 2020). This molecule may also signal fresh blood for feeding in the lampreys.

Glycine is a common gustatory stimulant for teleosts, such as in zebrafish (Oike et al. 2007), channel catfish (Caprio 1978; Kanwal and Caprio 1983; Ogawa and Caprio 2010), sea catfish (*Arius felis*) (Michel and Caprio 1991), rainbow trout (Jones 1990; Kohbara and Caprio 2001), and the Persian sturgeon (A*cipenser persicus*) (Shamushaki et al. 2007). While we found glycine to have stimulatory effects on the sea lamprey’s pharynx, it did not stimulate the larval brook lamprey’s pharynx (Baatrup 1985) or solitary chemoreceptor cells located on the outer surface of the gill vent in adult brook lampreys (Baatrup and Døving 1985), but it was stimulatory to solitary chemosensory cells in sea lamprey gill vents (Daghfous et al. 2020). This suggests that the responses we observed may be due to solitary chemosensory cells, but this is unlikely as newly transformed lampreys lack solitary chemoreceptor cell abundance on gill vents (Suntres et al. 2020) and these cells were not seen in the scanning electron microscope preparations of the pharynx that were examined in this study. Additionally, we observed receptor cell activity responses to l-arginine, which did not stimulate SCCs in adult sea lampreys (Daghfous et al. 2020), suggesting that the responses observed in our study are likely not attributed to SCCs. Interestingly, glycine was also found to be a key chemical compound contributing to the taste of sea food commonly consumed by humans (Fuke and Kônosu 1991). These authors found that glycine was required in extract solutions to maintain the characteristic taste of abalone, sea urchin, snow crab and scallops. Glycine, which is found and contributes to the characteristic taste of sea foods, may be an important positive prey indicator especially for sea lampreys and other predatory fish species.

Our results show that TDCA has a strong stimulatory effect in all pharyngeal regions, which may be indicative of the important function that bile acids serve in appraising food. For example, the gall bladder of well fed and older rainbow trout contains high levels of bile acids (Brant et al. 2013), thus bile acids may signal food quality in lampreys. Bile acids are digestive surfactants that are also important biological signaling molecules for fish including lampreys (Buchinger et al. 2014). Bile acid pheromones, petromyzonol sulfate and allocholic acid released by larval sea lampreys stimulate olfactory mediated attractive responses by adult sea lampreys during upstream spawning migration (Bjerselius et al. 2000). While the bile acid TDCA stimulates gill pore solitary chemosensory cell responses in adult sea lampreys (Daghfous et al. 2020), the current study is the first test of a bile acid as a tastant in the gustatory system of a lamprey. Electrophysiological evidence for gustatory detection of bile acids was also seen in teleost fish such as rainbow trout (Yamashita et al. 2006) and the channel catfish (Rolen and Caprio 2008). Behavioral investigation shows that bile acids are aversive tastants in the teleost fish, silver dollar *(Metynnis argenteus*), Nile tilapia (*Oreochromis niloticus*), green swordtail (*Xiphophorus hellerii*), and roach (*Rutilus rutilus*), but are attractive to Mexican cavefish (*Astyanax fasciatus*), which feeds on feces (Kasumyan and Vinogradskaya 2019). Though our results provide support for bile acids as tastants for sea lampreys, future behavioral studies are needed to determine whether bile acids elicit aversive or attractive feeding behaviors on juvenile lampreys.

Baatrup’s (1985) study on taste responses from larval brook lampreys, showed responses following applications of the amino acids arginine, l-alanine and l-serine, but not to glycine or l-proline. In our study of juvenile sea lamprey, we found that glycine is a tastant detected by sea lampreys. One possible explanation for this discrepancy is the different developmental stage used between the studies, where we tested on recently metamorphosized sea lampreys, while Baatrup (1985) focused on larval brook lampreys. Another possible explanation are the diets between the two species following metamorphosis. For example, related sympatric fish species, the carnivorous kutum (*Rutilus frisii kutum*) and omnivorous roach (*Rutilus rutilus*) were shown to have opposite taste preferences for sucrose, acetic acid and NaCl, indicating that closely related fish species with differing diets can have different behavioral responses to the same tastants (Goli et al. 2015).

Unfortunately, there are no studies on lamprey gustatory systems outside of the sea and brook lamprey. Work on other predatory species and non-predatory species can give insight into which gustatory functions are conserved across lampreys and parse out which features are specific to diet, as well as further our understanding of the functional evolutionary origins of the gustatory system. Additionally, elucidation of the neural pathways and organization of taste afferents will be needed to allow for selective recording of taste fibers and to understand the behavioral roles of tastants in lampreys.

## Data Availability

I will upload any supplemental data that is required.
